# Synthesis, Characterization and Safety Evaluation of Sericin-Based Hydrogels for Controlled Delivery of Acyclovir

**DOI:** 10.3390/ph14030234

**Published:** 2021-03-08

**Authors:** Moawia M. Al-Tabakha, Shujaat Ali Khan, Akram Ashames, Hamid Ullah, Kaleem Ullah, Ghulam Murtaza, Nageeb Hassan

**Affiliations:** 1 College of Pharmacy and Health Sciences, Ajman University, P.O. Box 346, Ajman, United Arab Emirates; m.altabakha@ajman.ac.ae (M.M.A.-T.); n.hassan@ajman.ac.ae (N.H.); 2 Center of Medical and Bio-Allied Health Sciences Research, Ajman University, P.O. Box 346, Ajman, United Arab Emirates; 3 Department of Pharmacy, COMSATS University Islamabad, Abbottabad Campus, Abbottabad 22060, Pakistan; drshujatalikhan@cuiatd.edu.pk (S.A.K.); hamidkhan.gu@gmail.com (H.U.); 4 Hamdard Institute of Pharmaceutical Sciences, Hamdard University, Islamabad Campus, Islamabad 44000, Pakistan; drkaleem.razmian@gmail.com; 5 Department of Pharmacy, COMSATS University Islamabad, Lahore Campus, Lahore 54000, Pakistan

**Keywords:** free radical polymerization, monomer, grafting, swelling ratio, pH-responsive, kinetic models, oral tolerability

## Abstract

Conventional formulations of antiviral drug acyclovir have various limitations such as low bioavailability. The current study was aimed at developing polymeric matrices for the controlled delivery of acyclovir using sericin as polymer and acrylic acid (AA) as a monomer. The free radical polymerization technique was used for hydrogel formulation. Briefly, sericin was chemically cross-linked with acrylic acid. *N*′-*N*′-methylene bis-acrylamide (MBA) and ammonium persulfate (APS) were used as cross-linker and initiator, respectively. FTIR spectra showed that acyclovir was successfully loaded into sericin hydrogel. SEM micrographs revealed that the outer surface was solid-like and smooth. According to DSC thermograms, the developed polymeric network was thermally stable. Amorphous nature of acyclovir was observed in XRD. The pH of medium and reactants’ concentration affected swelling dynamics and acyclovir release pattern. In addition, drug release occurred through a diffusion-controlled process. Sericin hydrogel suspension was well tolerable up to 3800 mg/kg of rabbits’ body weight. Haematology and serum chemistry results were well within the range signifying normal liver and kidney functions. Similarly, histopathology slides of the rabbit’s vital organs were also in normal condition without causing any histopathological change. It was concluded from the findings that sericin-co-AA polymeric matrices are ideal for the pH-dependent delivery of acyclovir.

## 1. Introduction

**Figure 1:**
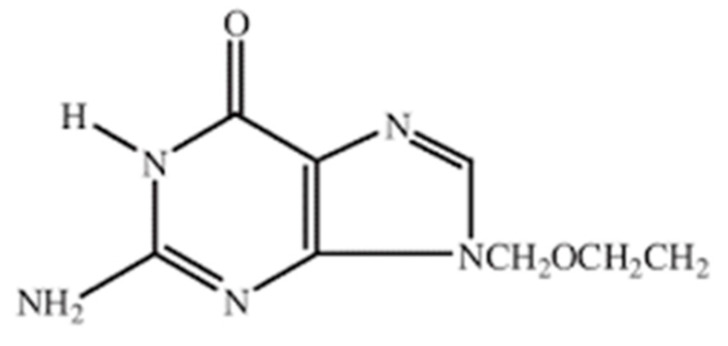
Structural unit of acyclovir.

Acyclovir (Figure 1) is a synthetic purine nucleotide analog having an antiviral effect [1]. It acts by binding with HSV-thymidine kinase [2]. It is generally used as a first-line treatment of herpes simplex, varicella-zoster, herpes zoster, and acute herpetic keratitis viruses [3]. Due to poor water-solubility (1.2 mg/mL) [4], short half-life (3 h) [5], poor percutaneous absorption [4], risk of thrombophlebitis after intravenous bolus administration [5], and low oral bioavailability (15–30%) [6], conventional tablets, intravenous formulations, and topical dosage forms of acyclovir are not only administered to patients in a high-frequency rate (5–6 times a day) [5] but also in large doses (200–800 mg) [4] to achieve desired therapeutic response, which is responsible for dose-dependent side effects [5]. Gastrointestinal absorption of acyclovir is carrier-mediated, thus its large doses saturate the carrier system. These undesired outcomes could be managed by developing a modified release system for acyclovir delivery. One of the strategies for modifying drug release is the development of hydrogel using a suitable polymeric system, such as sericin-based pH-responsive (SbpR) hydrogels. Since acyclovir absorption occurs in the small intestine, SbpR hydrogels can potentially contribute to the improved absorption and enhanced bioavailability of acyclovir by signifying pH-based swelling behavior and drug dissolution dynamics [3].

Polymeric hydrogels are the hydrophilic networks of polymers having three-dimensional structures with good mechanical strength [7]. On absorbing aqueous solution, they undergo swelling, likely due to the presence of hydrophilic groups [8]. Normally, the hydrogels have a solid-like appearance; however, the swollen hydrogels resemble soft rubber or living tissue [9]. There are certain drawbacks of conventional hydrogels. For instance, neither their morphology is homogenous, nor are they mechanically strong. Besides, their response to stimuli and swellability at equilibrium is also poor [10]. There are several strategies to resolve these limitations. Chemical modification of polymer is one of those approaches, which are widely used for improving the features of hydrogels [11]. The resulting hydrogels exhibit necessary swelling dynamics, promising drug loading efficiency, excellent mechanical strength, enzymatic resistance, biocompatibility, biodegradability, and non-toxicity. In addition, physical and chemical cross-links are formed between the polymeric chains, which make the hydrogels water-insoluble [12]. These features of hydrogels suggest their valuable use for controlled drug delivery.

Researchers have a great interest in using natural polymers for developing biocompatible, biodegradable, and non-toxic hydrogels [7]. Sericin is one of the promising natural polymers used in biomedical and drug delivery systems [13]. Bombyx mori (silkworms) produces sericin protein whose texture resembles glue [13]. Its molecular weights range from 20 to 400 kDa. Sericin contains eighteen amino acids comprising essential amino acids, especially serine (32%). The total contents of hydroxy amino acids in sericin are approximately 46%. It contains about 42% of polar amino acids and 12% of non-polar amino acids. Its polar groups (hydroxyl, amino, and carboxyl) of amino acid side chains contribute in cross-linking and copolymerization of sericin with other polymers/monomers [14]. The structural formula of sericin comprises random coil and β-sheets. Its sol-gel transition is noted under the effect of temperature, moisture, and/or mechanical stretching, which changes sericin coil structure for β-sheets [15]. The literature search reveals the inhibitory effect of sericin on cancer, bacterial growth, oxidative stress, coagulation, and tyrosinase [16,17,18]. Sericin is a hydrophilic material with a capability to maintain a moist environment of the wound [19,20,21] and absorb wound exudates. Its hydrophilic behavior is due to the presence of high contents of hydrophilic amino acids. Moreover, sericin enhances mammalian cell proliferation [22]. Sericin is prone to the action of proteolytic enzymes present in the body and, hence, it is biodegradable [15]. It is biocompatible and a natural resource for developing new protein-based materials [14]. Sericin can be cross-linked or copolymerized with other materials [23]. Several previous studies have revealed the scaffolds and nanocomposites of sericin, aiming at their use for wound healing [13,24,25,26,27,28]. Sericin-based scaffolds and nanocomposites showed an accelerated wound healing effect.

Acrylic acid, also named propenoic acid, is a commonly used hydrophilic, bioadhesive, the mechanically strong, and pH-responsive monomer for designing hydrogels with pH-dependent features [29]. It is the simplest unsaturated carboxylic acid having a vinyl group linked with a carboxylic acid terminus and, thus, can be polymerized via polymerization reaction (e.g., free radical polymerization) to obtain a polymer [30]. Acrylic acid and its esters have a potential of combining with themselves to produce polyacrylic acid or other monomers or polymer by acting at their double bond site, producing copolymers or homopolymers that are further used in different fields [10]. Acrylic acid grafting endows pH-responsiveness to the fabricated system, which could be employed as a vehicle for modified drug release [31]. Furthermore, the initiators are the species that are used to start the process of polymerization. A commonly used initiator is ammonium persulfate (APS) [29] that was used in this study. Moreover, *N*′,*N*′-methylene bis-acrylamide (MBA) was employed as a cross-linker, which is the most important component of hydrogel that converts water-soluble polymers to insolubilized polymeric hydrogels [32,33,34,35,36,37,38,39,40,41].

This study was designed for the development of sericin-based hydrogels having a potential of the pH-dependent release of acyclovir and to study its safety profile. The free radical polymerization approach was used for the development of a series of formulations by varying the feed ratio of sericin, acrylic acid, and MBA. Chemically, the fabricated hydrogels comprised non-covalent (ionic) grafting of acrylic acid on to polymeric chains of sericin through its functional groups. carboxyl groups of sericin backbone were utilized for graft polymerization through ionic bonds with hydroxyl groups of acrylic acid monomer. This monomer is also responsible for the pH-sensitive behavior of the prepared hydrogels that allows acyclovir release at a specific target site resulting in improved bioavailability. Therefore, this study reports conjugation of sericin with acrylic acid to get hydrogel having anticipated features. This study describes the optimization of formulation parameters and composition to get sericin hydrogels with varied cross-linking densities, good mechanical strength, pH-based swelling dynamics, and controlled drug release behavior.

## 2. Results and Discussion

In this study, nine formulations of sericin-co-AA hydrogel’s were formulated by varying concentrations of polymer (SR1, SR2, SR3), monomer (AC1, AC2, AC3), and cross-linker (MB1, MB2, MB3) were used. Hydrogels have been synthesized through the method of free radical polymerization. Sericin, Acrylic Acid, MBA, and APS has been used as polymer, monomer, cross-linker, and initiator, respectively.

### 2.1. Physical Appearance

The synthesized hydrogels had a smooth and transparent appearance. After drying, the color of some hydrogels was changed to a yellowish and some to a dark yellowish color due to variant polymer concentrations. Polymerization of sericin and AA resulted in stable hydrogels. The variable concentration of polymer and monomer affected the transparency of hydrogels. Low polymer resulted in hydrogels of a light yellowish color and a high concentration gave a golden yellow color to the hydrogels. A similar effect of polymer concentration on hydrogel color was also reported by Hu and Deng [37] while studying silk sericin-g poly(acrylic acid/attapulgite) composite superabsorbent. Hydrogels with a higher monomer and polymer concentration were more sticky and elastic. A higher concentration of cross-linker resulted in hydrogels having excellent stability and strength. All formulations retained their shape in swelled form and exhibited good gelling properties. These results can be correlated to the findings of Ullah et al. [33] while investigating chitosan-AMPS-AA pH-responsive hydrogels for oxaliplatin delivery to the colon.

### 2.2. Sol-Gel Analysis

**Figure 2:**
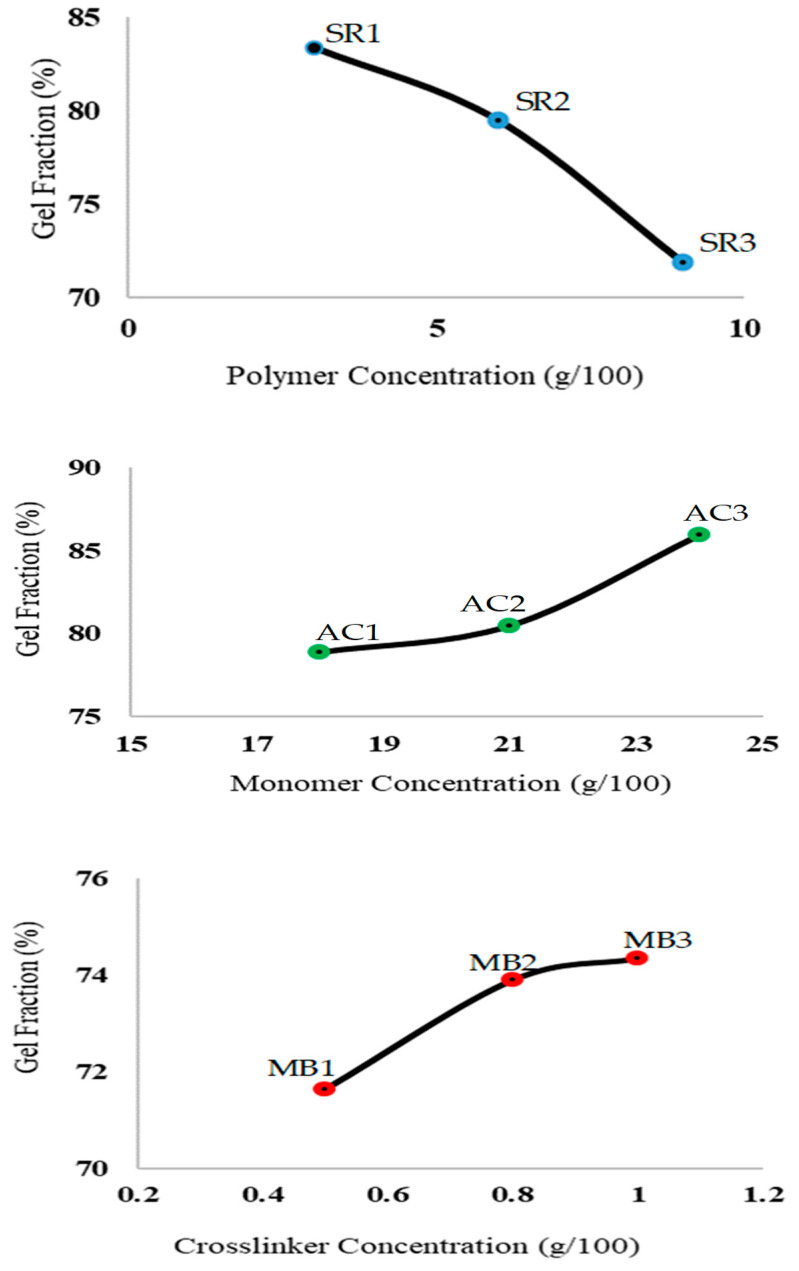
Sol-gel fraction findings of various formulations.

A sol-gel analysis was used to know about the cross-linked and un-cross-linked proportion of the reactants. The effect of the different ratios of sericin, AA, and MBA on the gel fraction is shown in Figure 2. The gel fraction significantly decreased with an increase in the sericin concentration; SR1-SR3 (*p* < 0.003), AA; AC1-AC3 (*p* < 0.000) and MBA; MB1-MB3 (*p* < 0.001). Hu and Dung synthesized silk sericin-g poly(acrylic acid/attapulgite) composite super absorbent and reported a similar effect of sericin on gel fraction [37]. Ullah et al. synthesized chitosan-AMPS-AA pH-responsive hydrogels for colonic release of oxaliplatin and reported a significant increase in gel fraction with a rise in the concentration of AA [34].

### 2.3. Determination of Drug Loading Efficiency (DLE, %)

Diffusion process was used for the assessment of acyclovir entrapment efficiency that was considerably affected by the feed ratios of polymers and a cross-linking agent. By increasing the sericin’s feed ratios, drug entrapment efficiency was increased, likely due to an increase in swelling hydrogel, while MBA affected acyclovir entrapment efficiency inversely (Table 1).

### 2.4. pH-Sensitivity, Swelling, and Drug Release

The key factors, such as the structure, content, and pH of the medium, control the swelling and release of drugs from the polymer matrix. Similarly, the ultimate release of drugs from the hydrogel, the structure and swelling behavior of the polymer system, the solubility of the contact between the loaded substance and the drug-polymeric network play a significant role.

Swelling studies of all formulations were conducted at pH 1.2, 6.8, and 7.4 to know and interpret the changes in swelling behavior with an increase in pH and with different concentrations of hydrogel components, i.e., monomer, polymer, and cross-linker.

**Figure 3:**
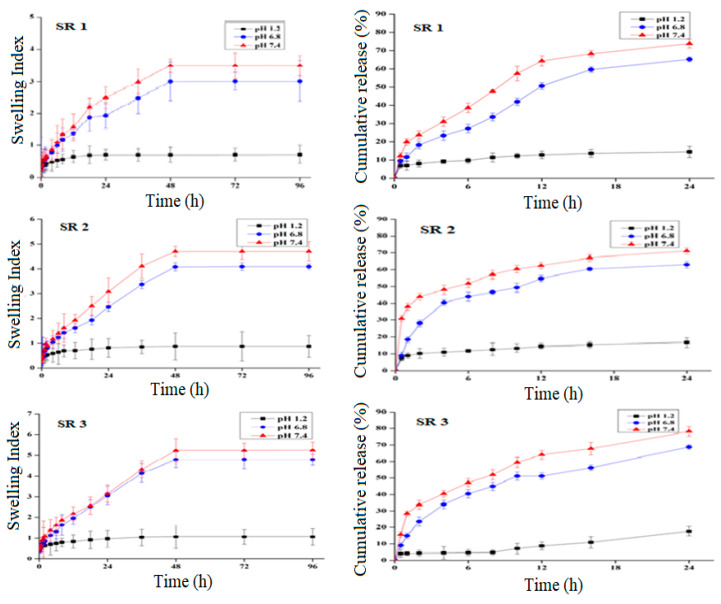
Swelling ratio and percentage drug release of formulation SR1-SR3.

According to the findings acquired from swelling and release studies (Figure 3), there was a significant difference (*p* < 0.05) between swelling index and drug release performance at low pH (pH 1.2) and high pH (pH 7.4). Each formulation provided a considerable release at pH 7.4. It has been proved from the result that the prepared formulations were pH-sensitive, owing to which a significant difference (*p* < 0.05) in swelling and release behavior at pH 1.2 and 7.4 was observed.

**Figure 4:**
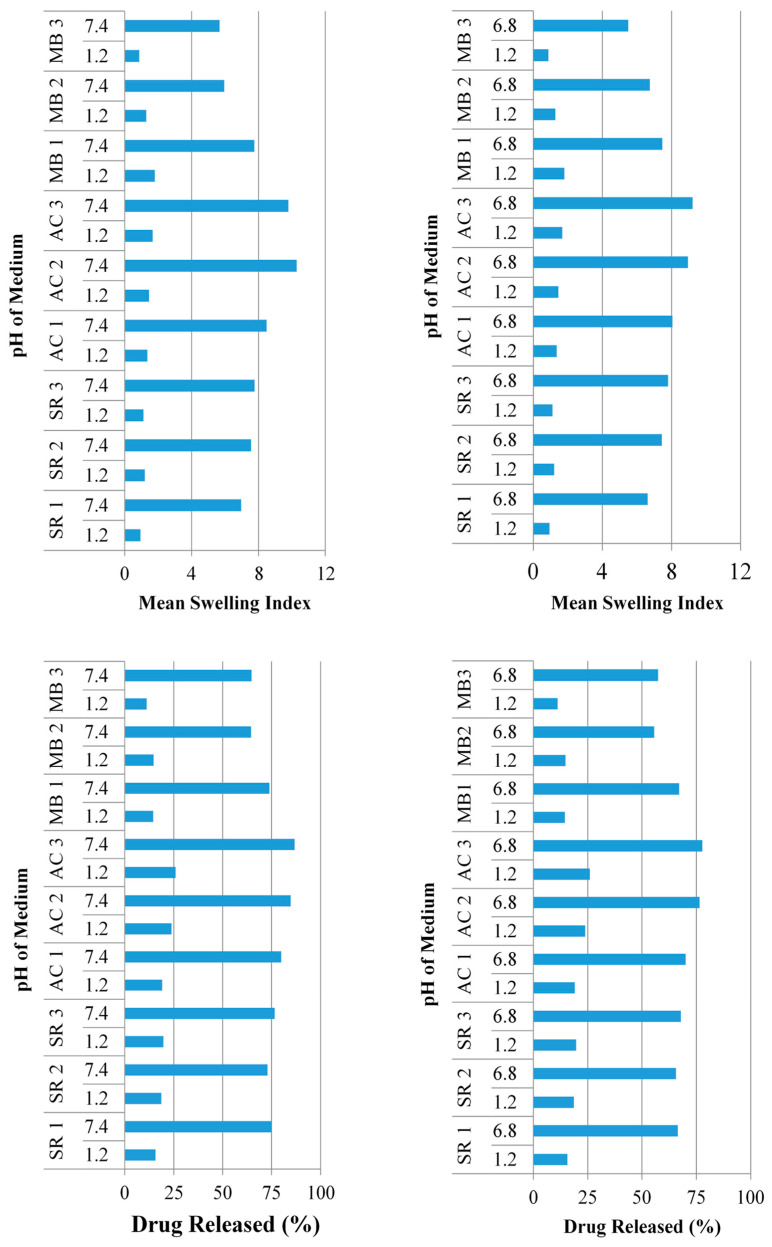
Comparison among in-vitro swelling dynamics and release behavior of all formulations at pH 1.2, 6.8, and 7.4.

Formulations with an increased feed ratio of sericin exhibited a considerable increase in swelling index (Figure 4) at pH 1.2 (*p* = 0.089), 6.8 (*p* = 0.004), and 7.4 (*p* = 0.001). The drug release pattern was identical to the swelling behavior, presenting considerable increase in drug release at pH 1.2 (*p* = 0.003), 6.8 (*p* < 0.000), and 7.4 (*p* < 0.001).

**Figure 5:**
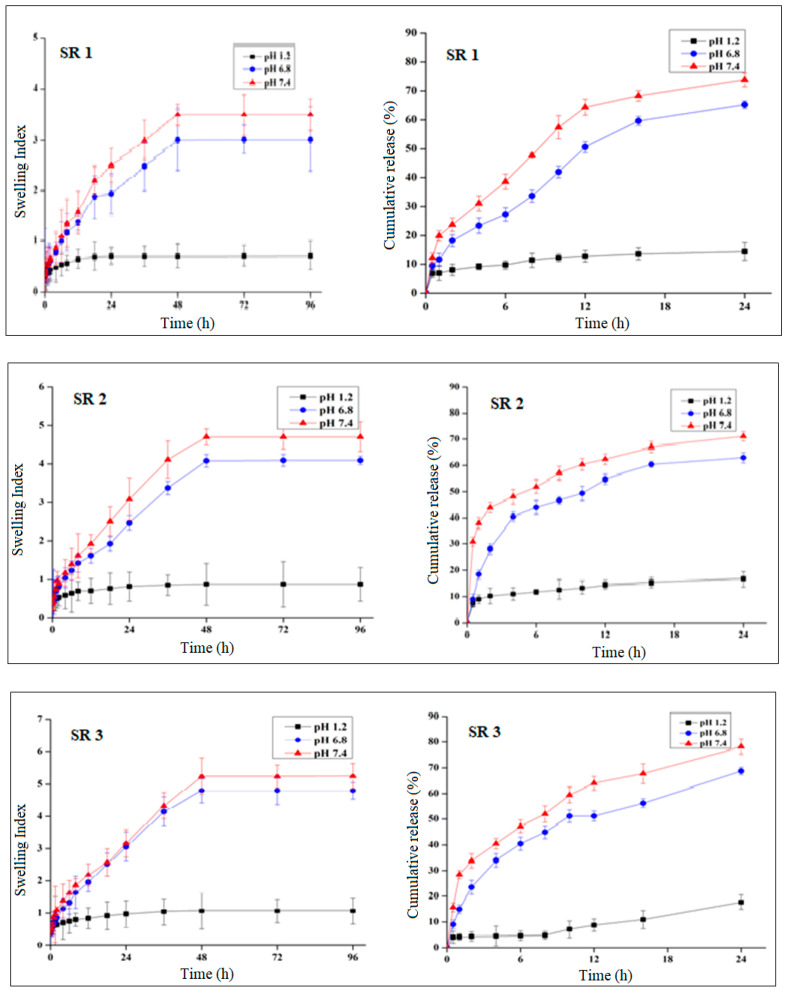
Swelling ratio and percentage drug release of formulation AC1-AC3.

Formulations with increasing feed ratios of AA (AC1-AC3) resulted in an increased swelling (Figure 5) at pH 1.2 (*p* = 0.01), 6.8 (*p* = 0.009), and 7.4 (*p* = 0.006). In the same way, drug release was significantly improved at pH 1.2 (*p* < 0.001), 6.8 (*p* = 0.001), and 7.4 (*p* = 0.001).

**Figure 6:**
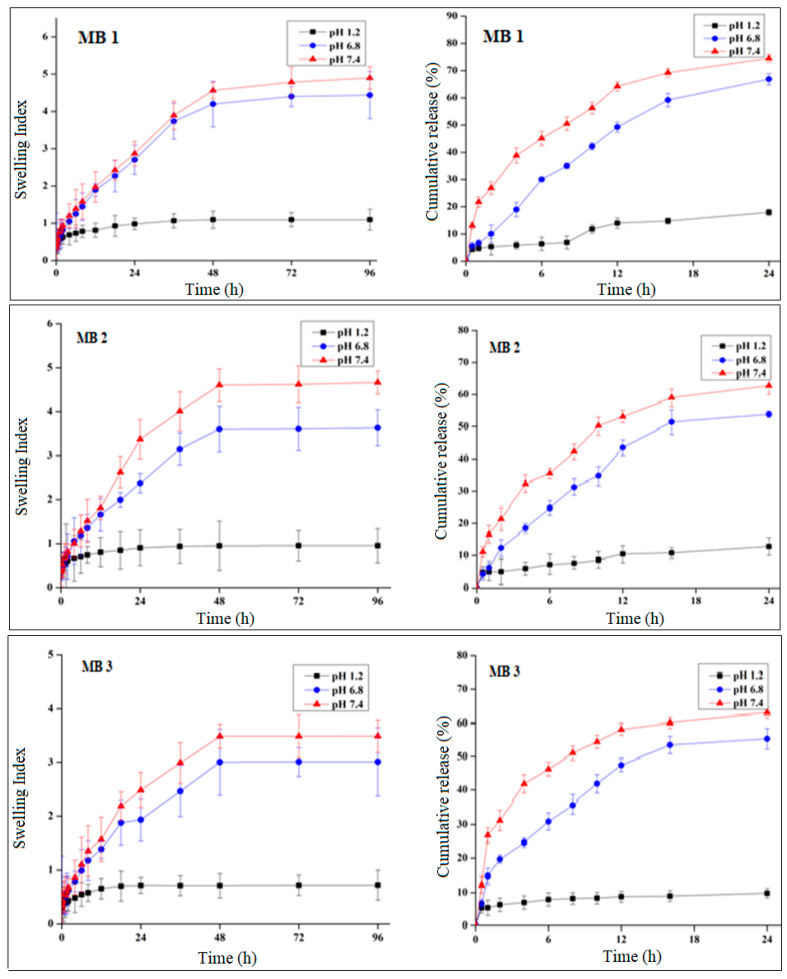
Swelling ratio and percentage drug release of formulation MB1-MB3.

The swelling index of the formulations (MB1-MB3) was extensively decreased at pH 1.2 (*p* = 0.003), 6.8 (*p* < 0.000), and 7.4 (*p* < 0.001) (Figure 6). Following the swelling, there was also major decline in drug release at pH 1.2 (*p* < 0.000), 6.8 (*p* < 0.001), and 7.4 (*p* < 0.003).

#### 2.4.1. pH Effect on Swelling and Drug Release

The resultant hydrogels displayed a high swelling index at basic pH (6.8 and 7.4) as compared to acidic pH 1.2. The mechanism by which swelling occurred is the deprotonation of anionic functional groups at higher pH, which brought negative group charge. At higher pH, negative medium ion repelled the functional group of the negative charge holding. This repulsion has caused the hydrogel disks to swell. In the case of sericin, the carboxylic group deprotonation happened at a high pH. The negative COO-repelled OH- of the medium resulted in the hydrogel swelling [15].

Similarly, the nearby pH and acidic polymer constituent markedly influence the behavior of the hydrogels in swelling and drug release. Effective swelling is accomplished when the pKa value of the buffer constituent is greater than the pKa value of the gels -COOH groups. Under these conditions, the gel will ionize, and the buffer will accept protons.

As drug release was highly dependent on swelling, a maximum release at pH 7.4 was provided by the disks that showed maximum swelling. Disks were unchanged at lower pH, which resulted in the low release of drugs. Remarkable variations in drug release were seen as evident from tables at pH 1.2 and pH 7.4.

#### 2.4.2. Effect of Polymer Concentration on Swelling and Drug Release

The result shows that swelling behavior was increased as we increased the sericin concentration. Shah et al. synthesized the triple-component nanocomposite (chitosan-silver-sericin) films [13] for antibacterial potential and reported that the expanded swelling of the composite network was related to the presence of free hydrophilic groups (–COOH, –NH2, and –OH) and to the strong interaction of the polymeric blend components, in particular the formation of hydrogen bond interactions.

According to another report, the increased sericin could also be related to the enhanced ionization resulting from the zero net negative charges on sericin [38].

The increased concentration of sericin exhibited a higher swelling ratio; that is why they entrapped a greater amount of the drug and released the drug more efficiently. Shah et al. reported a similar effect [13]; that the increased sericin concentration increases the release with an increase in sericin and may also be linked to the hydrophilic nature of sericin with polar functional (hydroxyl, amino, and carboxyl) groups initiating rapid sericin dissolution in water contact.

#### 2.4.3. Effect of Monomer Concentration on Swelling and Drug Release

According to Ullah et al. [33], the increase in swelling and release of the drug with increased AA content was owing to the influence of more ionizable groups leading to relaxation of the polymer chain.

Ranjha et al. prepared pH-sensitive hydrogels of chitosan-co-acrylic acid for controlled delivery of verapamil using MBA as cross-linker and AA as monomer and reported that the swelling ratio increased with the increasing quantity of AA [29].

By increasing the AA concentration, the swelling behavior of gel was increased; as a result, more drugs were released. This behavior can be credited to the existence of hydroxyl (–COOH) groups in the AA structure. Ullah et al. reported similar actions of swelling ratio and drug release by varying the monomer concentration as the swelling index was extensively increased at pH 7.4 with an increase in AA concentration [34]. This comparable effect was shown by the drug release also as an increase in AA concentration results in increased drug release at pH 6.8 and 7.4.

#### 2.4.4. Effect of Cross-Linker Concentration on Swelling and Drug Release

According to the given result, as we increased the cross-linker concentration the swelling index decreased and ultimately gave less drug release.

The explanation behind the decreasing swelling ratio, drug entrapment, and percent release of drugs due to MBA was the higher cross-linking ratio that reduced mesh size in a polymeric matrix. The overall trend resulted in a decreased swelling ratio with greater cross-linker concentration. These results can be connected to the previous work performed by Malik et al. [3] who synthesized pH-sensitive hydrogel for controlled release to manage ulcerative colitis. According to the same report by Khalid et al. [35], the increase in MBA raised the entanglement among monomer and polymer and impeded protonation and eventually decreased swelling and the release of drugs.

Furthermore, different models for investigating the pattern of drug release were applied on dissolution data. We have seen that the kinetic R2 value of the first-order release is near to 1, which proved that order and mechanism of drug release followed first-order release kinetics. Higuchi model’s R2 values for all formulations were greater than 0.5, showing that the mechanism of drug release supported diffusion base model [36].

Koresemeyer–Peppas kinetics was also used to evaluate the mechanism of drug release [34]. The “*n*” value of all formulations is below 0.5, which indicates that the drug follows non-Fickian release independent of variable polymers, monomer, and cross-linker concentration [35].

### 2.5. Chemical Characterizations

#### 2.5.1. Fourier Transform Infrared Spectroscopy (FTIR)

**Figure 7:**
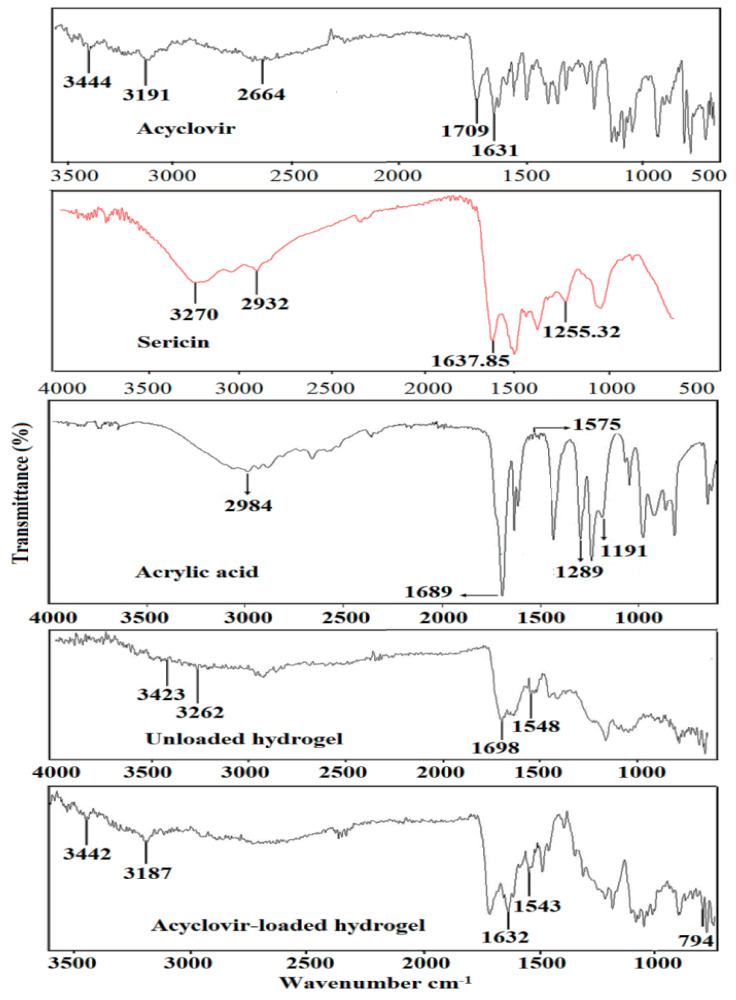
FTIR spectral graphs of Acyclovir, Sericin, AA, Unloaded hydrogel, and ACY-loaded hydrogel.

The FTIR spectra of acyclovir, sericin, acrylic acid, unloaded, and the drug-loaded hydrogels are shown in Figure 7. The IR-spectra of the active drug showed peaks at 3444, 3191, 2664, 1709, and 1631 cm^−1^. Sericin showed peaks at 3270 cm^−1^ and other characteristic peaks occurring at 2932, 1637, 1511, and 1255 cm^−1^. IR-spectra of AA displayed peaks at 2984, 1689, and 1575, the further two peaks present at 1289 and 1191 cm^−1^. In the case of unloaded hydrogels, peaks were observed at 3423, 3262, 1698, and 1548 cm^−1^ and drug-loaded hydrogels showed various peaks at 1632, 1543, and 794 cm^−1^.

In general, FTIR spectral analysis is used to verify the graft and chemical nature of the newly synthesized hydrogels and the separate components [3]. Few main peaks observed in FTIR spectra of acyclovir at 3444, 3191, 2664, 1709, and 1631cm^−1^ represent *N*–H stretching vibrations, O–H stretching vibrations, C–H bond stretching movements, C=O stretching, and bending of *N*–H, respectively. These peaks can be linked to FTIR result of the study reported by Malik et al. [3] while investigating acyclovir loaded cross-linked β cyclodextrin and carboxymethyl cellulose hydrogels. The sericin spectrum was characterized as; a broad spectrum visible in 3272 cm^−1^ range was caused by both N–H and O–H vibration, 2933 cm^−1^ can be certified to C–H bond stretching, the peak present at 1653, 1511, 1252 cm^−1^ was assigned to N–H bond vibration of amide I, amide II, and amide III respectively. Similar peaks were reported by Shah et al. [13] while investigating the healing of the wound and antibacterial activity of (chitosan-silver-sericin) films for moxifloxacin.

In AA, peak that absorbed are present at: 2984 cm^−1^ is dictated to (O–H stretching), 1689 cm^−1^ represent (C=O stretching), 1575 cm^−1^ is attributed to (C=O bending of carboxylic group). The other two bands present at 1289 and 1191 cm^−1^ are allocated to (C–C stretching) and (C–O stretching), respectively. These peaks are in close accord with the FTIR results explained by Ullah et al. [34] while synthesizing gelatin-based hydrogel for delivery of oxaliplatin to the colon.

FTIR spectra of hydrogel formulated system give an absorption peak at 3423 cm^−1^ which indicate both the stretching vibration of *N*–H and O–H bonds of sericin. Another strong peak seen at 1543–1698 cm^−1^ can be assigned to one of the groups of sericin. These results are in close connection with the study of Hu and Deng [37] while investigating the swelling behavior of silk sericin-g poly (acrylic acid/attapulgite) hydrogel.

Similarly, the peak present at 1159 cm^−1^ (C–O stretching) of AA was shifted to 1182 in unloaded and drug-loaded hydrogel, respectively. Such a similar report is given in Ullah et al. [33] while studying colonic delivery of oxaliplatin through hydrogel.

The distinctive peaks of acyclovir at 1712 and 1629 cm^−1^, showing the (*N*–H bending and C=O stretching), were found at 1711 and 1632 cm^−1^, respectively, in acyclovir-loaded hydrogels while these peaks were not present in unloaded hydrogels, which confirms the successful entrapment of the drug. Malik et al. synthesized β cyclodextrin and carboxymethyl cellulose hydrogels for acyclovir and reported similar peaks of acyclovir in loaded hydrogel [3].

#### 2.5.2. X-rays Diffraction Analysis (XRD)

**Figure 8:**
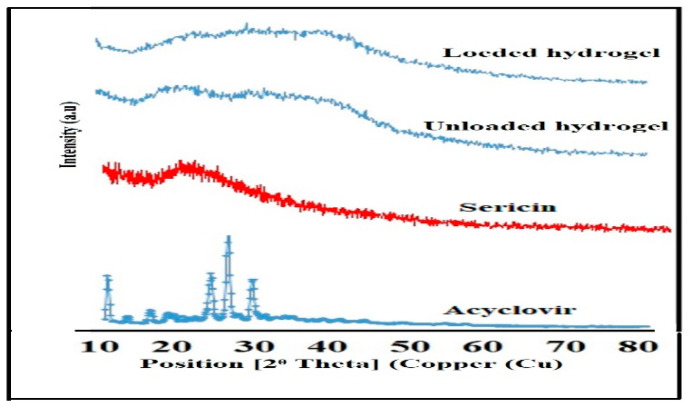
XRD model of Acyclovir, Sericin, unloaded, and drug-loaded hydrogels.

The XRD of acyclovir (Figure 8) at 2θ is 21.45°, 25°, and 32.20° characterize its crystallinity. Some other peaks were observed at 2θ = 10.59°, 23.39°, 22.9°, and 29.60°. Pure sericin demonstrates quality broad diffraction peak at 2θ = 20.8°. The unloaded hydrogels exhibited a weak peak at 2θ = 22°. No sharp peaks are observed for loaded hydrogel.

Generally, XRD analysis is performed to investigate the behavior of reactants and graft polymeric matrix. They may show crystal or amorphous nature [3]. The sharp and major peaks in the XRD diffractogram of acyclovir at 2θ = 22.50°, 25°, and 29.9° signify its crystalline nature. Sohail et al. synthesized polyvinylpyrrolidone and 2-acrylamide-2-methylpropane sulphuric acid-based cross-linked matrix for acyclovir and reported similar distinctive peaks signifying crystallinity of acyclovir [36]. Pure sericin showed a distinctive broad peak at 2θ = 21.1°, which represents the crystalline state of sericin. Shah et al. reported a similar effect [13] while studying the wound healing and antibacterial potential of chitosan-silver-sericin films for moxifloxacin. The XRD pattern of unloaded hydrogel shows a weak peak at 2θ = 21 indicating that the crystalline nature of sericin was decreased after polymerization. The result is according to the work done by Sohail et al. [36] while studying the controlled release new polyvinyl pyrrolidone and 2 acrylamido 2 methylepropane sulphunic acid-based cross-linked hydrogel for acyclovir. Furthermore, the diffractogram of ACY-loaded hydrogel showed large diversity from individual polymer, drug, and the unloaded hydrogel. A sharp peak of acyclovir was absent representing that the drug is dispersed within the matrix system. The results are in agreement with the recent report conducted by Sohail et al. [36].

#### 2.5.3. Scanning Electron Microscopy (SEM)

The hydrogel’s microstructure morphology is considered among the most significant features affecting overall swelling and drug release.

**Figure 9:**
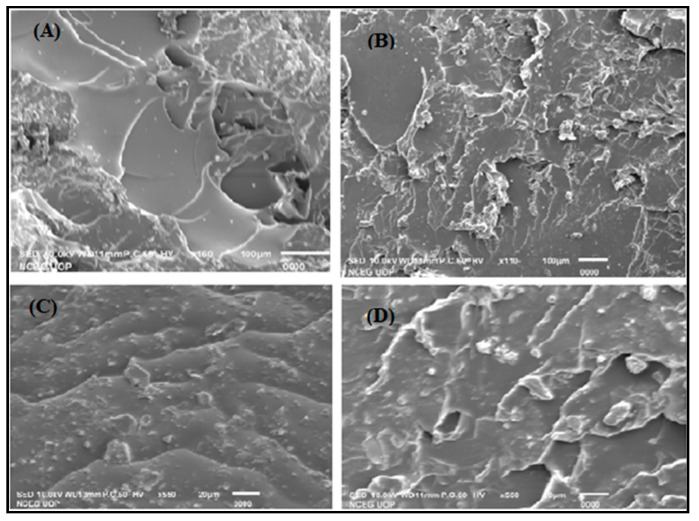
SEM microstructures of surface morphology of unloaded and loaded hydrogels (**A** and **C**) and cross-sectional morphology of un-loaded and loaded hydrogels (**B** and **D**).

Figure 9A displays SEM micrographs of the unloaded hydrogel’s outside area with pores at a certain range from each other that indicate a porous surface of the newly formed polymeric matrix. The cross-sectional view (Figure 9B) showed a soft glassy outside view with some cracks and sharp edges.

The surface of the acyclovir-loaded hydrogels (Figure 9C) showed the loss of some pores and a decline in size as well. Similarly, Figure 9D shows the internal broken view of the ACY-loaded hydrogel, which is non-porous with some cracks and crinkle on the surface and pointed edges.

The surface view of the unloaded hydrogel may be examined to display circular pit morphology. These pits can help in drug loading. The broken surface of the unloaded hydrogel had layers with spiky edges of glassy appearance. The surface view of acyclovir-loaded hydrogel seems to have small spherical pits, which prove that acyclovir was entrapped into the hydrogel. The cross-sectional photomicrographs of acyclovir-loaded hydrogel showed rough, wrinkly type appearance with little cracks, which differentiate it from unloaded hydrogels. This change in structure or morphology of loaded hydrogels may be due to the drug loading. According to another report, the porous shape allows water molecules to pass into the hydrogel system, so they provide an area for water permeation and produced interface sites for media or any buffers. The porous structure is also the primary explanation of why the hydrogels have higher swelling index values [2].

#### 2.5.4. Differential Scanning Calorimetry (DSC)

**Figure 10:**
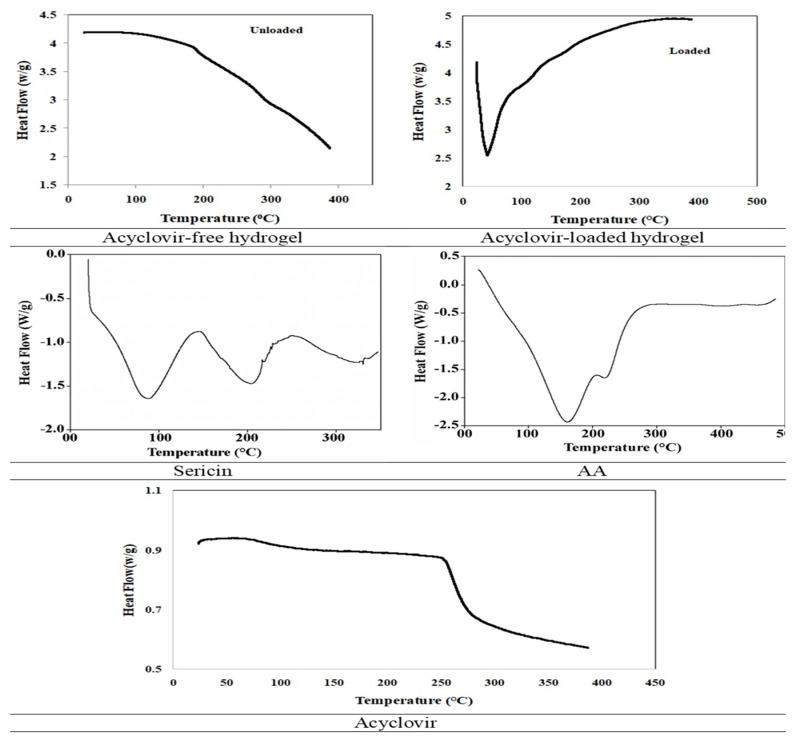
Differential Scanning Calorimetry (DSC) thermograms of various samples.

Acyclovir displayed a major endothermic peak at 265.91 °C (Figure 10). Two analytical broad and prominent endotherms were shown, earlier at 87.8 °C and later at 210 °C, in the analysis DSC for pure sericin exploration. The AA displayed two endothermal peaks. The initial one was in the range of 100–160 °C and the latter in the range of 200–225 °C. In loaded and un-loaded hydrogels, no sharp peak was observed at a lower temperature.

Acyclovir showed a major endothermic peak at 265.8 °C. Peaks observed at the very first stage are related to loss of water while peaks observed at a final stage in DSC thermogram represent either their melting point or oxidative degradation. The results have similarities with a study conducted by Malik et al. [3] where β-cyclodextrin and carboxymethyl cellulose hydrogels for controlled drug delivery of acyclovir were synthesized.

According to DSC examination, pure sericin displayed two investigative large and significant endotherms, earlier at 88.2 °C, indicating moisture loss, and later at 211 °C, linking to the thermally induced movement of the molecules and also cause peptides bonds melting of sericin with an increased thermal disintegration. This similar effect is in agreement with the study conducted by Zhang et al. [39] while investigating the fabrication of silk sericin with electrospinning method.

In an acrylic acid situation, two endothermic peaks were observed; the former was in the 100–150 °C range and the later one was found in the 200–230 °C range. Peaks observed at the first stage corresponded to the loss of water while the last peaks encountered for the melting or oxidative decomposition. This result was in agreement with the report, examined by Ullah et al. [33] while synthesizing hydrogel as a potential drug carrier. Where no peaks in unloaded and loaded hydrogels were found at a lower temperature, it is concluded that our formulations were rigid and stable at high temperature [38].

### 2.6. Release Kinetics

To assess the kinetic release of hydrogels, different models have been practiced. Table 2 shows that “R2” values of the first order for all formulations were observed to higher than the values of zero order. This suggests that the release of the drug depends on concentration. In case of the Higuchi model, the “R2” for all the formulations were greater than 0.45, which suggests that the release mechanism was diffusion-based, which is well supported by the results of swelling studies where a higher polymer network relaxation was noted. The “*n*” values of Koresemeyer–Peppas model for the majority of the formulations were higher than 0.45, which indicates non-Fickian release while in the case of pectin-LA-MAA hydrogels Fickian release was observed as the values were below 0.45.

### 2.7. Oral Tolerability and Safety Profiling

#### 2.7.1. Determination of the Maximum Tolerance Dose (MTD)

Sericin-co-AA hydrogel dispersion in 200–4000 mg/kg of body weight was administered through oral route in separate doses. The rabbits showed different symptoms such as unusual breathing, tachycardia, excessive urine excretion, and appetite loss at the dose of 4000 mg/kg of body weight, probably because of a full stomach. No death or any illness condition appeared during MTD study. This allowed us to claim that the MTD for Sericin-co-AA hydrogel formulation was estimated to be 3800 mg/kg body weight.

#### 2.7.2. Monitoring of the General Conditions

For any abnormal condition appearance, the rabbits were kept in regular observation during the experimental duration. It was experiential that the selective MTDs were tolerated well by the experimental subjects (rabbits), as all symptoms appeared to be normal in both the groups exclusive of any illness or death condition.

#### 2.7.3. Serum Chemistry and Haematological Profiles

Orally administered hydrogel suspension might cause inappropriate problems because of the unreacted contents, which may be filtered out from hydrogels. That is why the hematological investigation was conducted; to find any abnormal condition comparatively with the control group. Serum chemistry was carried out for the observation of kidney and liver functions. The results are given in Table 3 and Table 4. The concentration of metabolites of the experimental group was seen to be in normal range compared with that of the control group. According to serum chemistry results, both liver and kidney groups’ performance were normal with no considerable differences.

#### 2.7.4. Histopathological Investigations

**Figure 11:**
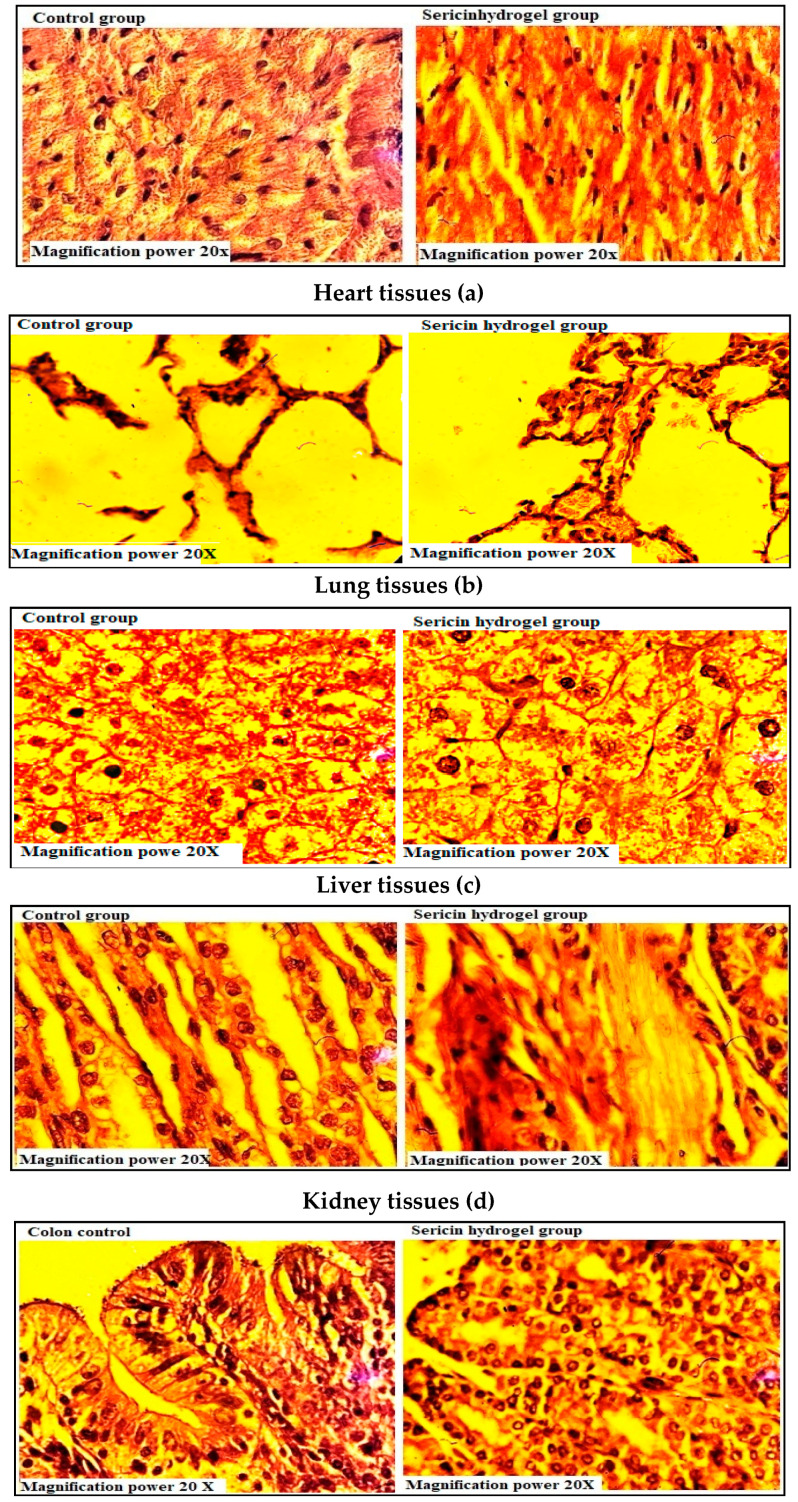
Histological examination of rabbit tissues of control and treatment groups (20× magnification power); (**a**) Heart tissues; (**b**) Lung tissues; (**c**) Liver tissues; (**d**) Kidney tissues.

The histopathological micrograms of the control and treatment groups of heart, lung, liver, kidney, and intestines are presented in Figure 11. Figure 11a illustrates the micrographs of the heart of the control and the treatment groups (sericin hydrogels). The cardiac myocytes were in normal order as well as the pericardium; myocardium and endocardium were found normal. There were no signs of hypertrophy in the cardiac muscles. As shown in Figure 11b, no alveolar or bronchial damage was observed in the treatment groups compared with the control one. Bronchus was found clear without permeation of the inflammatory cells. Similarly, the cilia were also found clear. According to Figure 11c, there was no difference in the overall histological micrographs of the control and treatment groups. The kidneys were found to be small in size (Figure 11d). No signs of bleeding existed and the nephrons were obvious. The glomerulus had defined space in the surrounding area as well. Furthermore, there is no evidence of necrosis, bleeding, or degeneration of the bowel mucosa (Figure 11e). There was no difference compared to the control group in the overall histological micrographs.

The serum chemistry and hematology results were well within the usual range showing no variation compared with the control group. Similarly, the histopathological analysis also showed a normal appearance. No gross histopathological changes were noted between the control and treatment groups. Our results were well support by the previous findings of Ullah et al. [40] while studying chitosan-based hydrogels for polyoxometalate and conducted oral toxicity studies in rabbits. Similarly, our results were also in close relationship with the previous study performed by Barkat et al. [41] while examining the synthesis PEG-based hydrogel for controlled delivery of oxiplatin (OXP) and carried out safety evaluation studies in rabbits.

### 2.8. Comparison of Sericin Based Hydrogels with Other Delivery Techniques for Acyclovir

Free radical polymerization is one the most widely used techniques in the synthesis of responsive hydrogels with profound pH sensitivity, swelling, and drug release characteristics. There is no need of specific temperature or pressure conditions and can be easily performed in an aqueous medium. Furthermore, the reaction is usually fast, and the synthesized gels are permanently cross-linked. Cost effectiveness and method simplicity are also responsible for its extensive applications in the synthesis of hydrogels for drug delivery.

Table 5 compares our newly developed sericin hydrogels with other delivery techniques used for acyclovir. The comparison was made on the basis of drug entrapment efficiency, amount of drug released, and time required for maximum drug release. Based on the said parameters, it is clear from the table that the properties of hydrogels prepared in this study are comparable with most of the previously reported formulations.

## 3. Materials and Methods

### 3.1. Chemicals

Acyclovir (99.8%) was purchased from Hangzhou Jinlan Pharm-Drugs Technology Co., Ltd., Hangzhou, China. Acrylic acid (AA) of Daejung, Korea, Silk sericin of Huizhou Xintiansi Biotech, Huizhou, China, Ammonium persulfate (APS), ethanol and *N*,*N*’-methylene bis-acrylamide (MBA) of Merck, Darmstadt, Germany, and HCl, KCl, NaOH, and KH2PO4 of Sigma Aldrich, Ayrshire, UK were purchased through local vendors.

### 3.2. Synthesis of Hydrogel

**Figure 12:**
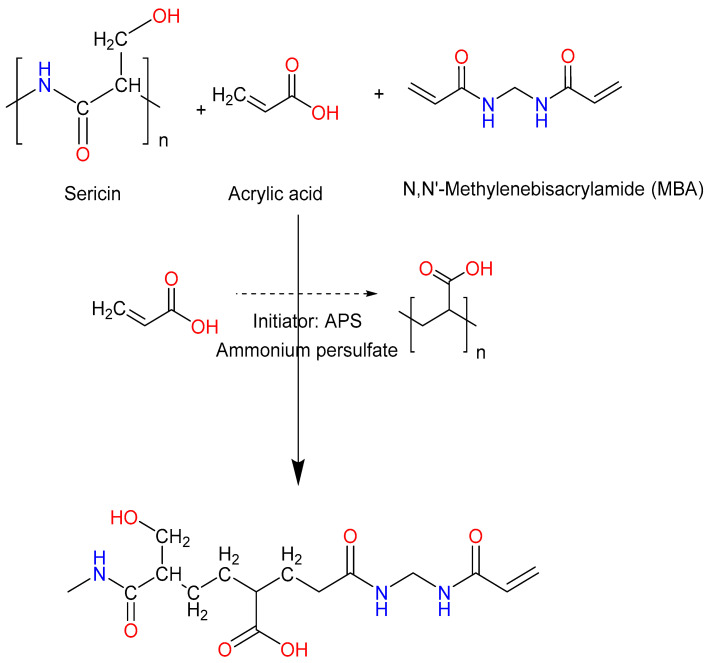
Diagrammatic illustration of ammonium persulfate (APS)-initiated graft copolymerization of acrylic acid onto sericin in the presence of MBA and an anticipated structure of the cross-linked network of the hydrogel.

Nine pH-responsive gel formulations (Table 6) were prepared through free radical polymerization approach (Figure 12) as previously reported [33] after minor modification. In brief, pre-weighed polymer (sericin or SR), monomer (AA), initiator (APS), and cross-linker (MBA) were dissolved in their respective solvents resulting in three separate solutions. The initiator solution was carefully added to acrylic acid and kept on stirring for 45 min by using a hot-plate magnetic stirrer (MS300HS, TOPS, Peshawar, Pakistan). After this, the cross-linking solution was poured into it carefully. The final weight was kept at 100 g by adding distilled water. The mixture was left on stirring for 45 min to get a homogeneous mixture. The obtained solution was poured in the pre-labeled 8 mm glass test tubes, enclosed in aluminium foil, and kept in a water bath (WB20, Polyscience, Hong Kong, China). The temperature was initially set at 40 °C and gradually increased to 55 °C. On the next day, after the entire night incubation process, glassy gels were formed. The gels were removed from the test tubes, washed with water–ethanol (1:1 *v/v*) combination, and cut into 8 mm discs. Initially, the discs were kept in room temperature to become dried and then were placed in an oven at 45 °C for the whole night until they gained their constant weight. The finally dried discs were piled up in glass containers at 37 °C. Some of the unwashed gels were kept in glass vials from each formulation for sol-gel analysis.

### 3.3. Chemical characterizations

#### 3.3.1. Fourier Transform Infrared Spectroscopy (FTIR)

The spectral matching approach was used to identify any possible drug, polymer, and monomer interactions. Before the test, 15 mg of unloaded and loaded hydrogels and raw material samples were ground using pestle and mortar and then passed through mesh 40 to achieve a uniform particle size and mounted on a spike. Scanning was done in the range of approximately 500–4000 cm^−1^. For analysis, FTIR spectrophotometer (Tensor-25 series, Ettlingen, Germany) was used and OPTUS software was used for the collection of data [33].

#### 3.3.2. X-ray Diffraction (XRD)

The main purpose of this procedure was to inspect the effect of cross-linking on the crystallinity of raw material and drug after loading in the hydrogel. The instrument was set by Cu-radiation (λ 1.534 Å) and applied 40 Kv of voltage. The analysis was done in the 10–100 ° angle range at 2θ with a rising speed of 2 °C/min [34].

#### 3.3.3. Scanning Electron Microscopy (SEM)

The shape, exterior, and cross-sectional morphological properties of the unloaded and drug-loaded hydrogels were evaluated by scanning electron microscope. For better conductivity, the samples were gold-platted and then fixed on the aluminum stub by using double adhesive tape. The instrument (Hitachi S-2460 N, Tokyo, Japan) was used for SEM analysis. By varying the magnification power, micrographs of different magnification powers were taken [35].

#### 3.3.4. Differential Scanning Calorimetry (DSC)

The melting temperature (Tm) and glass transition (Tg) of drug, polymer, monomer, unloaded, and drug-loaded hydrogels were analyzed through DSC. We took 5 mg grounded sample wrapped in an aluminum pan and sealed using a punch and die. One pan was placed in the heating chamber by selecting the standard temperature mode and another one was used as a reference. The heat was given in the range of 20–500 with the acceleration of 10 °C/min while maintained 50 mL/min nitrogen flow. Universal Analysis 2000 (version 4.5) software was used for data collection [33].

### 3.4. Sol-Gel Analysis

The sol-gel study was performed to find out the efficiency of cross-linking and the ratio of unreacted to reacted contents. The weighed, unwashed, dried hydrogel disks were attached to soxhlet apparatus carrying deionized boiling water. Boiling was continued for 4 h, followed by disc removal and oven-drying at 45 °C. Drying was continued until a constant weight was obtained. The analysis was performed in triplicate.

Sol fraction (%) = [w° − w_ext_ ÷ w°] × 100 (1)

Gel fraction (%) = 100 − Sol fraction (2)

where w° is the dry hydrogel weight before the extraction process and wext is the hydrogel weight after the extraction procedure [3].

### 3.5. Drug Loading

The method of swelling and diffusion was used to load acyclovir. In phosphate buffer pH 7.4, 100 mg of acyclovir was dissolved. At 37 °C, dried weighed hydrogel disks were immersed in 100 ml of acyclovir solution. Disc position was changed every 12 h. This process was continued for 72 h. The discs were taken off from acyclovir solution, blotted with filter paper, and subjected to drying at 40 °C until a constant weight was attained [13].

### 3.6. Estimation of Acyclovir Content

The quantity of acyclovir loaded in each hydrogel was determined by solvent extraction method. This method involves extracting acyclovir from the polymeric network. The extraction was done in the phosphate buffer pH 7.4, which was used for drug testing. After 24 h, the buffer was replaced with a fresh buffer. This cycle continued until no more acyclovir was left in the buffer. Acyclovir entrapment amount was calculated from the absorbance value obtained by using UV–Vis-spectrophotometer (T80+, Pg instrument, UK) [35].

### 3.7. Swelling Study

To determine the swelling activity and pH sensitivity of the hydrogel, pre-weighed (Md), oven-dried hydrogels were incubated at pH 1.2, 6.8, and 7.4 at 37 °C in 100 mL buffer medium. The swollen hydrogels were removed from the containers at different time points; the excess solvent was cleaned off using filter paper, weighed (Ms), and transferred to the respective containers. The procedure proceeded until the hydrogels attained constant weight. During the analysis, the pH of the buffer medium was maintained on regular basis. The swelling index was determined by the following formula:

Swelling index (Q) = Ms/Md × 100 (3)

where “Ms” indicates the weight of the swelled hydrogel disc, “Md” is the initial weight of the dried hydrogel disc [33].

### 3.8. In-vitro Drug Release Study

Drug release activity of hydrogels was studied using a dissolution medium (900 mL) of 0.1 M HCl (pH 1.2) and 0.2 M phosphate buffer pH (6.8 and 7.4) in USP dissolution apparatus-II (Microtech digital) at 75 rpm and 37 °C. At specific time intervals, 5 mL medium was removed and examined by using UV–Vis spectrophotometer (T80+, Pg instrument, UK) at a λmax 255 nm. To maintain sink condition during the experiment, 5 mL of fresh medium was added [33]. Various kinetic models, including zero order, first order, Higuchi, and Koresemeyer-Peppas models were used to find out the order and mechanism of drug release from the polymeric network. The selection of the best model is based on the closeness of “R2” value to 1.

### 3.9. Oral Tolerability and Safety Profiling

The study was thoroughly conducted under the guidelines given by the Food and Drug Administration Authority (FDA, 20050 and Laboratory Animal Science research facility (2009)) [13]. The protocol of the study was seriously evaluated and moral approval was approved by the Research Ethics Committee (REC), COMSATS University Islamabad, (Abbottabad campus) Pakistan (Ethical approval notification; Phm. Eth/FA17-CS-M10/18-010-74). For oral safety evaluation of the hydrogel suspension, the maximum tolerance dose (MTD) method was conducted. For calculation the MTD, four healthy rabbits (two male and two female) of 1–2 kg were chosen. The hydrogel in suspension form was orally given to rabbits in a 200–4200 mg/kg range in a separated dose. For the determination of oral tolerability and safety assessment, six rabbits (three male and three female) were chosen. The rabbits were separated into two groups, Group-1 (control) and Group-11 (hydrogel suspension). Separate cages were provided for rabbits’ housing and before dosing, all the rabbits were kept in fasting condition up to 15 hrs with water ad libitum. To the control group, normal saline was given. According to MTD, 4000 mg/kg of body weight hydrogel suspension was given in divided form for seven days. The sign of salivation, urination, diarrhoea and faeces, tremor, and illness were critically monitored during this duration. On the last day of the experiment, samples of blood were assembled from these two groups and kept in ethylenediaminetetraacetic acid (EDTA) tubes for the biochemical and haematology examination. The samples were sent to Ali labs (Islamabad) and their investigations were done by a consultant pathologist.

### 3.10. Hematology and Serum Chemistry

Various haematology tests were carried out to find out the numbers of white blood cells (WBCs), red blood cells (RBCs), platelets, basophils, eosinophils, haemoglobin, packed cell volume (PCV), and mean cell volume (MCV). For the examination of all these parameters, automated haematology analyzer (Fernwald, Germany) was used. For protein, globulin, albumin, amylase, alkaline phosphatase, aspartate and alanine transferase, potassium, glucose, creatinine, magnesium, cholesterol, bilirubin, and urea levels were determined by using fully automated chemistry analyzer [36].

### 3.11. Histopathological Investigations

To determine the histopathology of the organ, a small piece of tissue from each organ was placed in histopathology cassette and then treated with absolute alcohol for 2 h to remove water. After that, the dehydrated tissues were soaked by sinking twice in paraffin wax at 54–60 °C for 45 min. At room temperature, the tissue was cooled down by inserting it in a paper boat. Afterwards, microtomy was done and the tissue was put in a water bath. An adhesive was applied on a glass slide and dipped in a water bath for adherence of ribbon to the slide. Each slide was placed for 3 min in xylol and then absolute alcohol. After that, the slides were bare for 2 min to the methylated spirit and followed by washing in running water for 1 min. The slides were stained for 3–5 min using Harris hematoxylin and washed for 15 s in running water. To remove the extra dye, the slides were exposed for 15 s to 1% acid alcohol. Each slide was again washed for 30 s in running water, followed by repeated exposure to ammonia solution until the blue color was attained by the tissue. The slides were treated with water for 2–3 min and then exposed for 2–3 min to counterstain eosin followed by washing for 30 s in running water. To dry the samples, alcohol was used in increasing concentrations. Finally, a drop of Canada balsam was put on each slide and enclosed with a coverslip. The slides were dried by air and then observed under a microscope [33].

### 3.12. Statistical Analysis

The data is described in triplicate as the Mean ± Standard Deviation (SD). To determine the statistical significance of sol-gel, swelling, drug loading, in-vitro drug release, and cytotoxicity data, T-test (two samples paired for the mean) was used while ANOVA was used for multiple group comparisons. *p*-value ≤0.05 was statistically significant.

## 4. Conclusions

In the current research, nine hydrogel formulations were successfully prepared using various combinations of sericin and acrylic acid using free radical polymerization technique. Herein, the novelty of this project was based upon the novel combination of polymer and monomer, which are not reported in the literature so far. The oral tolerability, hematology, and histopathology studies of the formulations are reported for the first time as well as we have tested formulation for in-vivo analysis in rabbits. In the case of sericin-co-acrylic acid hydrogels, post-synthesis characterization confirmed the positive cross-linking (FTIR), thermal stability (TGA/DSC), decreased crystallinity of the prepared hydrogels after cross-linking and drug loading (XRD), and rough surface morphology (SEM). The swelling, drug loading efficiency, and degree of drug release were accurately regulated by changing the polymer ratio. The synthesized hydrogel also had sensitivity to the pH of the medium, and the degree of the hydrogel’s swelling and drug release can be controlled by a change in media pH. In vitro drug release verified the formulations followed the pattern of the Higuchi model, which suggested the diffusion-based release of the drug. Koresemeyer–Peppas’ value of “*n*” indicated that all formulations based on the non-Fickian process.

## Figures and Tables

**Table 1:** The entrapment efficiency of the developed formulations.

Formulation Code	Entrapped Acyclovir (mg)/Hydrogel Disc ± SEM
SR-1	62.4 ± 0.606
SR-2	64.5 ± 0.548
SR-3	65.8 ± 0.404
AC-1	69.3 ± 0.669
AC-2	72.4 ± 0.721
AC-3	73.8 ± 0.721
MB-1	59.4 ± 1.270
MB-2	56.2 ± 1.334
MB-3	52.1 ± 1.228

**Table 2:** Drug release kinetic models of all formulation of hydrogels having a variable concentration of sericin, acrylic acid, and MBA.

Formulations	pH	R^2^				Value of “*n*”
		Zero-Order	First-Order	Higuchi	Koresemayer–Peppas	
SR-1	1.2	0.673	0.699	0.88	0.454	0.388
	6.8	0.923	0.966	0.976	0.650	0.75
	7.4	0.855	0.939	0.976	0.574	0.731
SR-2	1.2	0.646	0.674	0.857	0.413	0.393
	6.8	0.747	0.848	0.940	0.573	0.728
	7.4	0.632	0.807	0.856	0.312	0.530
SR-3	1.2	0.901	0.911	0.914	0.661	0.510
	6.8	0.934	0.982	0.974	0.800	0.901
	7.4	0.825	0.935	0.978	0.543	0.713
AC-1	1.2	0.911	0.910	0.796	0.519	0.373
	6.8	0.843	0.940	0.986	0.568	0.656
	7.4	0.813	0.949	0.971	0.427	0.572
AC-2	1.2	0.594	0.637	0.792	0.274	0.323
	6.8	0.683	0.861	0.893	0.298	0.474
	7.4	0.404	0.613	0.661	0.204	0.415
AC-3	1.2	0.983	0.951	0.951	0.714	0.576
	6.8	0.829	0.937	0.978	0.618	0.732
	7.4	0.753	0.907	0.949	0.481	0.643
MB-1	1.2	0.638	0.658	0.827	0.432	0.359
	6.8	0.888	0.954	0.984	0.651	0.765
	7.4	0.767	0.955	0.950	0.547	0.721
MB-2	1.2	0.833	0.849	0.944	0.569	0.408
	6.8	0.906	0.943	0.974	0.805	0.871
	7.4	0.833	0.911	0.978	0.577	0.709
MB-3	1.2	0.559	0.575	0.793	0.42	0.323
	6.8	0.847	0.906	0.977	0.646	0.746
	7.4	0.672	0.770	0.900	0.483	0.658

**Table 3:** Serum chemistry profile (hydrogel dispersion) of the control and treatment groups (*n* = 6 rabbits: 3 male rabbits and 3 female rabbits) for each.

Parameter	Group-I (Control)		Group-II (Treatment)	
	Male Rabbits (*n* = 3)	Female Rabbits (*n* = 3)	Male Rabbits (*n* = 3)	Female Rabbits (*n* = 3)
Total Protein (g/L)	72.37 ± 0.71	71.15 ± 0.53	74.12 ± 0.22	67.57 ± 0.40
Globulin (g/L)	21.42 ± 0.44	20.85 ± 1.14	22.26 ± 0.43	20.81 ± 0.12
Albumin (g/L)	54.43 ± 0.62	52.56 ± 1.61	56.66 ± 0.83	53.92 ± 0.43
ALT (U/L)	83.10 ± 1.81	81.21 ± 1.68	84.54 ± 0.58	82.34 ± 0.45
ALP (U/L)	135.07 ± 0.18	131.97 ± 1.63	136.91 ± 0.70	132.66 ± 0.75
AST (U/L)	58.99 ± 0.46	60.06 ± 0.70	61.44 ± 1.80	63.32 ± 0.34
Cholesterol (mmol/L)	109.87 ± 1.44	108.56 ± 0.80	106.74 ± 1.39	107.45 ± 1.40
Glucose (mmol/L)	8.63 ± 0.35	9.01 ± 0.44	7.99 ± 0.19	8.25 ± 0.97
Creatinine (µmol/L)	154.38 ± 2.13	157.26 ± 0.78	156.98 ± 0.38	158.32 ± 0.70
Urea (mmol/L)	16.08 ± 0.53	14.67 ± 0.86	16.12 ± 1.97	13.80 ± 0.86
Uric acid (mg/dL)	3.87 ± 0.13	3.91 ± 0.66	3.95 ± 0.75	3.93 ± 0.07
Magnesium (mmol/L)	0.86 ± 0.14	0.96 ± 0.44	1.12 ± 0.19	1.06 ± 0.12
Phosphorus (mmol/L)	2.82 ± 0.11	2.80 ± 0.86	2.45 ± 0.25	2.67 ± 0.17
Potassium (mmol/L)	5.99 ± 0.12	6.29 ± 0.26	6.34 ± 0.25	6.35 ± 0.05
Sodium (mmol/L)	159.62 ± 1.36	158.45 ± 0.36	155.60 ± 0.81	157.83 ± 0.45

**Table 4:** Hematological profile (hydrogel dispersion) of the control and treatment groups (*n* = 6 rabbits: 3 male rabbits and 3 female rabbits) for each.

Biochemical Analysis	Group-I (Control)		Group-II (Treatment)	
	Male Rabbits (*n* = 3)	Female Rabbits (*n* = 3)	Male Rabbits (*n* = 3)	Female Rabbits (*n* = 3)
pH	7.11 ± 0.07	7.01 ± 0.17	7.71 ± 0.13	7.50 ± 0.09
Hemoglobin (g/L)	114.33 ± 1.53	109.87 ± 0.78	112.87 ± 4.81	106.23 ± 1.18
RBCs × 10^12^/L	6.10 ± 0.12	6.00 ± 0.21	5.87 ± 0.09	6.04 ± 0.11
Eosinophils × 10^9^/L	8.30 ± 0.07	7.98 ± 0.16	8.24 ± 0.18	7.05 ± 0.73
WBCs × 10^9^/L	0.04 ± 0.01	0.04 ± 0.02	0.04 ± 0.01	0.04 ± 0.00
Lymphocytes × 10^9^/L	2.43 ± 0.06	2.38 ± 0.13	2.72 ± 0.18	2.52 ± 0.28
Neutrophils × 10^9^/L	4.11 ± 0.04	4.31± 0.71	4.41 ± 0.18	4.51 ± 0.27
Basophils × 10^9^/L	0.33 ± 0.05	0.29 ± 0.12	0.43 ± 0.02	0.39 ± 0.13
Platelets × 10^9^/L	354.50 ± 4.40	350.97 ± 1.74	356.80 ± 0.97	352.73 ± 1.71
PCV (L/L)	0.47 ± 0.01	0.44 ± 0.02	0.45 ± 0.02	0.46 ± 0.04
MCV (L/L)	66.84 ± 0.78	65.50 ± 0.93	67.77 ± 0.59	62.39 ± 0.79

**Table 5:** Comparison of sericin based hydrogels with other delivery techniques for acyclovir.

S. No.	Formulations	Intended Amount of Drug Loaded (mg)	Drug Loading (%)	Maximum Drug Release (%)	Time (h) for Maximum Drug Release	Reference
1	Solid Lipid nanoparticles	10	85	100	24	[42]
2	SR tablets	100	100	100	12	[43]
3	Thermo-responsive in-situ gels	600	98.15–99.75	97	6	[6]
4	Muco-adhesive nanoparticles	25	71	94	12	[44]
5	Gastro-retentive muco-adhesive microspheres	200	51.42−80.46	75	8	[45]
6	Sericin-based hydrogels	100	73.8	86.85	24	Present study

**Table 6:** Hydrogels prepared with different concentrations of sericin, acrylic acid (AA), and MBA.

Formulations	SR (g)/100 g	AA (g)/100 g	MBA (g)/100 g	APS (g)/100 g
SR-1	3	15	0.3	1
SR-2	6	15	0.3	1
SR-3	9	15	0.3	1
AC-1	9	18	0.3	1
AC-2	9	21	0.3	1
AC-3	9	24	0.3	1
MB-1	9	15	0.5	1
MB-2	9	15	0.8	1
MB-3	9	15	1.0	1

## Data Availability

The data presented in this study are available on request from the corresponding author. The data are not publicly available due to privacy or ethical restrictions.
